# Present and future of telemedicine for pediatric care: an Italian regional experience

**DOI:** 10.1186/s13052-023-01408-9

**Published:** 2023-01-17

**Authors:** Gianvincenzo Zuccotti, Valeria Calcaterra, Andrea Foppiani

**Affiliations:** 1 Department of Biomedical and Clinical Science, University of Milan, 20157 Milan, Italy; 2 Pediatric Department, Buzzi Children’s Hospital, ASST-Fatebenefratelli-Sacco, 20154 Milan, Italy; 3 Department of Internal Medicine, University of Pavia, 27100 Pavia, Italy; 4 International Center for the Assessment of Nutritional Status (ICANS), Department of Food, Environmental and Nutritional Sciences (DeFENS), University of Milan, 20133 Milan, Italy

**Keywords:** Telemedicine, Pediatric care, Regional experience, Telehome care, Children

## Abstract

Telemedicine has been recognized as an integral part of the National Health Service in Italy. Telemedicine can be adopted in the diagnostic therapeutic assistance pathway and in individual assistance plans. In the region of Lombardy, home care models from the perspective of the project of a public virtual hospital have been introduced. A regional operational center was proposed to ensure continuing care utilising organizational and technological solutions to deliver healthcare services remotely, with high quality standards, a positive economic impact and user friendly services for both the user and the professional. In the field of pediatrics telemedicine was also introduced at the Vittore Buzzi Children’ Hospital, in Milan, the capital of the region of Lombardy. These included routine pediatric hospital activities and innovative programs, such as early discharge, telecardiology, online supervised exercise training and preventive healthcare. Telehealth represents the evolution of health care delivery systems to adapt to new technology and the needs of the pediatric population, offering a strategic system to invest in children’s health.

The term telemedicine is used to refer to the provision and delivery of health-related services, which may include medical care, education of health care providers and patients, health information services, and self-care through telecommunication and digital communication technologies [1–3].

Telemedicine does not replace the traditional healthcare service concerning the doctor–patient relationship, but integrates it to improve effectiveness, efficiency and appropriateness. Telemedicine configures, itself as a tool to extend the traditional practice outside the usual physical spaces, complying with all rights and obligations inherent in any health care act [4].

According to the recent guidelines issued in 2020 by the Italian Ministry of Health, “telemedicine” has been recognized as an integral part of the services of the National Health Service [5]. Telemedicine can be adopted in the diagnostic therapeutic assistance pathway and in individual assistance plans [5]. Telemedicine, with integrated devices to monitor the patient’s health status, offers the possibility to reduce hospital admissions and to anticipate their discharge, with important psycho-social and economic repercussions [4, 5].

The incorporation of telemedicine in pediatric settings has been steadily increasing for the past two decades. Remotely delivered visits to children are particularly valuable, as patients and their families often have to deal with impediments, such as a limited number of pediatric specialists and barriers to long-distance travel [6–9]. As with adults, the pediatric telemedicine service can offer faster access and can help improve the quality of health care, increasing the usability of treatments, diagnostic services and remote medical advice, as well as enabling the constant monitoring of vital parameters, to reduce the risk of harm to people at risk or suffering from chronic disorders. Telemedicine also provides a centralized hub for a child’s health care to ensure continuity, and inequitable access to pediatric care [10]. It also gives us the chance to see children in their home environments which assists with other social information, such as the living environment, and the interactions with family and caregivers.

In the Lombardy region in the north of Italy, the need to implement telemedicine had already emerged before the COVID pandemia, to enhance assistance in the area, activating home care models from the perspective of the project of a public virtual hospital, to ensure continuous care utilising organizational and technological solutions that activate the delivery of healthcare services remotely, in total safety and with high quality standards with a positive economic management impact and user friendliness for the user and the professional.

During the Coronavirus disease 2019 (COVID-19) pandemic, an innovative telemedicine project involving the implementation of active domiciliary monitoring (named COD19) and a homecare hospital system (named COD20) in COVID-19 patients was proposed. A regional operational center was set up based on the Amazon Web Services (AWS) Serverless platform; it was created by the collaboration of the University of Milan, the Health Protection Agency (Agenzia per la Tutela della Salute, Health Protection Agency—ATS) Metropolitan City of Milan andthe Local Health and Welfare Agency (Azienda Socio-Sanitaria Territoriale—Local Health Authority, ASST Fatebenefratelli-Sacco hospitals), and industrial research partners [11]. This monitoring system provided a continuous assessment of critical medical conditions, offering the detection of socially and clinically relevant issues. This service was readily needed during the COVID-19 pandemic in response to the identification of comorbidities in the monitored patients; additionally, it provided an improvement in management after the lockdown. In fact, the COD20 system supported the integration between the hospital and the healthcare territorial system and automated and simplified the appointment booking process through an online connection with the regional booking center [11]. The use of telemedicine was also implemented for integrated management of post-acute COVID-19 syndrome in order to connect data, clinicians and patients, with an experience of integration between regional inter-hospital network and territory care, including hospitals serving different locations, from the city center to the outskirts and mountain settings.

Telemedicine was also introduced in the field of pediatrics at the Buzzi Children’ Hospital, Milan, the capital of the region of Lombardy, Italy. After the lesson learned from COVID-19, using the COD20 consultation system in pediatric hospital activities daily, remotely delivered patient visits were introduced in dietetic and nutritional counseling and in pneumological, gastroenterological, rheumatological and endocrinological visits and consultations. The patient–specialist video consultation service allowed clinicians to perform an assessment of clinical conditions and any need to visit the patient in person [11, 12].

More recently, in this hospital, three novel and interesting modalities of telemedicine were also offered to young patients. The first innovation was a project involving early discharge (72 hours before) with a home care visit, using a Tytocare® device that enables a healthcare provider to perform guided medical examinations. With this system, the doctors can safely observe children in a calm and comfortable environment while assessing their clinical condition without the influence of additional stressors. The early discharge represents a cornerstone of the optimization of care in the pediatric field, where, as envisaged by the “European Association for Children in Hospital Charter” [13], it is necessary that every hospitalized child is discharged as soon as possible to guarantee their psychophysical well-being. Economic impact of telehome care will also be evaluated. As reported, home hospitalization allows cost optimization, both structural and personnel expenses [14–17]; prospectively telehomecare in the pediatric field can cost up to 9% less than standard care [14].

A second telehealth proposal at Buzzi Children’ Hospital was a telecardiology program. This is a new, simple and safe method for carrying out and reporting electrocardiograms in children by moving the site of interaction between doctor and patient from the cardiology clinic to the web using innovative tools. This need derives from the desire to maintain the hospital’s high level of response to the requests of territorial pediatric services offering technological devices which allow, in total safety and with high quality standards, the carrying out of a “traditional” electrocardiogram in a more comfortable and usable form for the user. A telecardiology service will offer the advantage of reducing the use of and loads of hubs/hospitals and avoid visits to hospital through the pediatric medical filter/local medicine by selecting hospital access only for patients suspected of a cardiac pathology, while still offering the possibility of responding to a frequent need for the general practitioner to discriminate patients with a suspected cardiac pathology (palpitation, chest pain, arrhythmia, murmur, etc.) and remove waiting lists.

Finally, as a third proposal, an online supervised exercise training for children with obesity, useful for promoting physical activity, improving physical fitness and reducing ponderal excess, was proposed [12].

Pediatric home care, adopted in Milan, will be also implemented with integrated communication with territorial pediatricians and territorial health-care structures. Tele-expertise services provide an opportunity to facilitate and strengthen interdisciplinary collaboration and access medical expertise of uncertainties in diagnosis and therapeutic plans [18]. The interaction and cooperative stimulation between specialists is a prerequisite for encouraging shared decision-making, with sharing information with the patient being key to promoting compliance.

Telemedicine will find a specific role in several pediatric medical specialties to maintain constant follow up with chronic patients (such as in the case of diabetes, asthma, cardiac disease, neurological disorders, and endocrinopathies) and/or to perform a screening step, where, in a short time, children receive consultation and information on the need (e.g., skin manifestations, and orthopedic problems) to perform exams before an in-person visit.

Considering the improvement in treatments in children with medical complexities, populations with chronic illnesses and medical fragility will present a high priority for the use of health services and technological systems to support their multiple health needs [19]. A hybrid “home–hospital” approach could be proposed as perspective in the future of pediatric care for “vulnerable” children.

The potential to expand the role of pharmacist services using telemedicine will be also considered in a regional and national context; it has a promising role, to coordinate the drug therapy monitoring with the hospital [20], to enhance pharmacists’ interactions with residents and nurses within the nursing facility [21] and to facilitate continuous follow-up, early detection of drug-related problems, and the identification of new needs and improvement points in patients [18].

Technological innovation can contribute to the reorganization of health care, specifically by supporting the shift of the fulcrum of health care from the hospital to the community, through innovative care models centered on the citizen and by facilitating access to services in the national territory [4, 11, 22].

Telemedicine also offers the opportunity to shift from episodic health care to preventive and pro-active continuous healthcare. Even in the presence of screening programmes, the health system relies heavily on the individual to initiate the care process through the identification of relevant symptoms. This model, while being parsimonious on resources, is not optimal, as individuals often miss early signs of disease and only seek medical attention after disease progresses to a more severe symptomatic phase. Telemedicine can support the shift to a preventive model, providing cost-effective technology to monitor continuously and unobtrusively at-risk patients, through the installation of sensors in their homes or using wearable medical devices able to continuously monitor relevant health parameters. The full shift to a preventive model also has the potential of being cost-effective through reduction of disease incidence. This model is also part of telehealth proposals of Buzzi Children’ Hospital in the context of the National Recovery and Resilience Plan.

However, despite its attractiveness, the use of telemedicine still shows some barriers, including technological challenges, the integration of workflow, perceived usefulness, regulatory issues and costs for hospital services, including equipment and personal and dedicated connections, and for users, who require an Internet connection and a computer, tablet or smartphone to use this service. Additionally, for telemedicine the applicable legal regulations are insufficient, and the existing ones are not clear [23]; in what concerns healthcare and the practice of medicine there are no uniform regulations at the European level [23]. Thus, the adoption of this tool remains a challenge in public services [24, 25].

To maximize the potential of telemedicine and to eliminate the barriers to its use, future research and initiatives are necessary to improve the cost-effectiveness of telemedicine services, to support free access to the Internet, to develop dedicated guidelines regarding data protection safety, informed consent, and professional liability, and to adopt payment reform for telehealth services [26].

Telehealth represents the evolution of health care delivery systems to adapt to new technology and the needs of the pediatric population, offering a strategic system to invest in children’s health.

## Data Availability

not applicable.

## Abbreviation

COVID-19: Coronavirus disease 2019
